# Physical Modeling of Cross Wedge Rolling Limitations

**DOI:** 10.3390/ma13040867

**Published:** 2020-02-14

**Authors:** Łukasz Wójcik, Zbigniew Pater, Tomasz Bulzak, Janusz Tomczak

**Affiliations:** Mechanical Faculty, Lublin University of Technology, 36 Nadbystrzycka str, 20-618 Lublin, Poland; z.pater@pollub.pl (Z.P.); t.bulzak@pollub.pl (T.B.); j.tomczak@pollub.pl (J.T.)

**Keywords:** cross wedge rolling, physical modelling, plasticine

## Abstract

This article presents the results of model tests aiming to verify the possibility of applying commercial plasticine as a model material for modelling the limits to the cross-wedge rolling process. This study presents a comparison of the results of laboratory testing and physical modelling of cross-wedge rolling (CWR) processes. Commercial plasticine was the model material used in the research to model 50HS grade steel formed in 1150 °C. The model material was cooled to 0 °C, 5 °C, 10 °C, 12,5 °C, and 15 °C. Physical modelling of neckings and slippages is only possible when the plasticine is heated to 12.5 °C prior to forming. Commercial plasticine does not enable one to model the cracking process inside the rolled element.

## 1. Introduction

Cross-wedge rolling is the basic plastic-forming process in the production of axial-symmetric geometry. The rolling process is performed using wedge-shaped tools. This technology allows one to obtain a product of high quality and accuracy. This method can be employed to manufacture semi-finished products later subjected to machining. Compared to other plastic working technologies used for manufacturing axial-symmetric products, this method allows for obtaining 60% less material waste. [1].

**Figure 1:**
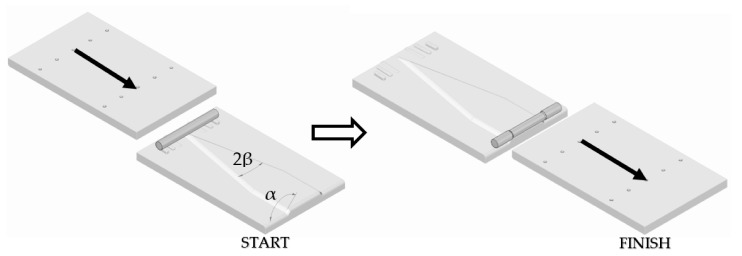
Scheme of the example cross-wedge rolling process.

Figure 1 presents an exemplary process of rolling axial-symmetric forgings.

The following limitations may occur in the process of cross-wedge rolling: uncontrolled slip of the formed material between the tools, necking of the formed step, and inner cracks in the formed product [2]. 

The above mentioned limitations occur usually as a result of an incorrect selection of geometrical parameters of the tools. 

The basic geometric parameters of tools used in the process of cross-wedge rolling are: α forming angle and β wedge angle of flare. These angles have a very significant influence on the cross-wedge rolling process and determine the stability of the process, values of the forces occurring during rolling, and cracking of the formed products. 

The values of the forming angle α ought to remain in the range 15°–40°. Increasing the value of this angle causes a significant increase in the axial force, which stretches the forging and, as a result, causes neckings to occur. In the case of the α angle, 15° inner crackings are more likely to occur in the formed product. 

β angle of flare ought to remain in the range 3°–15°. Increasing the value of the flare angle causes the tools to elongate, whereas exceeding 15° may cause uncontrolled slips to occur between the formed material and the tools [3].

According to Andreev et al. [4], overlapping on the surface of the formed product may occur when the value of the angle of flare exceeds 35°.

The β angle is calculated from dependency Equation (1), wherein the three remaining conditions ought to be fulfilled. The first condition is to determine a stable rolling process without slip, as shown in Equation (2). The second condition determines the limit value upon exceeding which necking of the sample will occur, as shown in Equation (3). The last condition pertains to the occurrence of crackings in the axial area of the formed product, as shown in Equation (4) [5].

(1) $$\sin\beta =0.009\frac{\mu }{\sin\alpha },$$

(2) $$\left(0.25+0.0038\alpha \right)\beta^{0.925}\leq 1.93,$$

(3) $$\frac{\sqrt{2\tan\alpha \tan\beta }}{\pi }\left(1+\frac{1}{\delta }\right)\left(\delta -1\right)\leq 0.2,$$

(4) $$\left(0.115+0.0038\alpha \right)\beta^{0.325}\geq 0.35÷0.4,$$

where: *α* is the forming angle; *β* is the wedge angle of flare; *µ* is the friction factor; *δ* is the deformation ratio; d_0_ is the diameter of the billet; and d is the diameter of the formed step.

Inner cracks in the rolling process are caused by the Mannesmann effect. The defects are usually longitudinal, axial cracks. The factors influencing the occurrence of the Mannesmann effect are [6,7,8,9,10]:

- cyclically interchanging compressive and tensile stresses,
- gradual damage of material cohesion resulting from low-cycle material fatigue.

In the investigation of the limits to the process of cross-wedge rolling, computer techniques [11,12,13] and, to a smaller extent, physical modelling [14] are used.

Physical modelling is a form of research on plastic forming of metals, in which the real material is substituted with model material. Model tests allow one to verify the theoretical observations concerning the investigated element, performing necessary tests in the laboratory conditions, obtaining results for further tests, limiting the testing time, and decreasing the research cost.

The material used for testing ought to fulfil the four main criteria of similarity to/wards the real material. In order for the simulation to be correct, it is necessary for the following aspects to be similar:

1. material flow curves for the model and real material,
2. friction conditions (it is suggested that the values of both friction factors and coefficients ought to be the same for both materials),
3. shape of the tools (scaling the dimension of the tools), and
4. forming kinematics of the investigated process.

Model materials used for physical testing were divided into two groups: metallic and non-metallic materials. The group of metallic materials consists of, among others: lead, sodium, Wood’s alloy etc., whereas the non-metallic group includes the following: plasticine, resins, natural and artificial waxes, cellulose etc. 

In the physical modelling tests, both the formed material and the tools are made of the model materials (polymers, wood, light metal alloys). The most commonly used methods for manufacturing the tools for physical modelling are mechanical processing and 3D printing. 3D printing allows one to manufacture tools of any shape from both polymer materials and metal powders. 

Physical modelling has been employed since the second half of the 20th century. Tests were performed using model materials usually at room temperature. At that time, physical modelling substituted the currently employed numerical simulations due to the fact that it was impossible to conduct calculations for complex cases of forming.

The majority of scientific papers on physical modelling were published in the 1980s by Japanese researchers. 

Chijiwa et al. [15] obtained and presented the results of plastometric tests that aimed to determine the applicability of plasticine as a model material for modelling the behaviour of hot-formed metals. After publishing the research results, plasticine became more frequently used in model testing.

Since then, many physical and laboratory tests on forging, extruding and rolling have been conducted. Physical modelling of forging processes were presented by, among others, Mandić [16] and Zhan [17], whereas Balasundar [18] and Khalili [19] introduced the results of physical testing of the extruding processes

The process of physical modelling of cross-wedge rolling was performed by Japanese researcher Danno et al. [20] who experimentally tested the phenomenon of torsion of the material during the process of rolling stepped shafts. In the 1990s, model testing of the cross-wedge rolling process was conducted by Dong et al. [21]. In their paper, the results of the research on the phenomenon of slipping between the tools and the billet, with lead as the model material. The results were compared to computer simulations using ANSYS/LS-DYNA.

The following papers on physical modelling of cross-wedge rolling were published in 2015–2019 by the research team of Lublin University of Technology.

In their study, Wójcik et al. [22] presented the results of the research on cross-wedge rolling of stepped shafts with plasticine at the temperatures of 0, 5, 10, 15, and 20 °C as the material model, whereas the tool material was ABS. The study compared the process of cross-wedge rolling of the real stepped shaft with the physical modelling process. 

Another paper describing physical modelling of cross-wedge rolling processes was published by Pater et al. [23]. Therein, the results of cross-wedge rolling of steel balls for mills were presented. Two rolling processes were investigated in the model form: cross-wedge rolling and skew rolling. The results were compared to those obtained for the real process. The real materials used were C45 and C60 grade steels, whereas the model material was plasticine cooled to 5 °C. The obtained results showed a significant convergence between the real and physical tests.

Upon analysing the model testing of the cross-wedge rolling process, it was observed that the authors did not concentrate on physical modelling of the limits (slipping, cracking, occurrence of neckings) occurring during the rolling process. It was therefore deemed beneficial to conduct laboratory and physical testing of the phenomena, limiting the cross-wedge rolling process.

## 2. Materials 

Model material used in laboratory testing was commercial plasticine PRIMO (Morocolor, Via Bassa Prima, Italy) of two types, called, in accordance with their colour, black and white. The applied plasticine is a non-metallic material. Apart from clay, oils and colouring pigments, it comprises mostly synthetic wax. 

Plastometric tests were conducted for the model material in the temperature range 0 °C to 20 °C by the research team Wójcik et al. [24]. On the basis of the results obtained in the research plastometric equations, described with the Formula (5) were designed. The constants describing the material are presented in Table 1.

(5) $$\sigma_{F}=C\varepsilon^{n_{1}}e^{n_{2}\varepsilon }{\dot{\varepsilon }}^{\left(m+bT\right)}e^{aT},$$

where: *σ_F_* represents flow stress, MPa; *ε* represents effective strain; $\dot{\varepsilon }$ represents strain rate, s^−1^; *T* represents temperature, °C; and *C*, *n_1_*, *n_2_*, *m*, *b*, *and a* are constant presented in Table 1.

Commercial plasticine aims to model the behaviour of 50HS grade steel formed at 1150 °C. The material model of 50HS grade steel was described by Formula (6).

(6) $$\sigma_{F}=C_{1}e^{\left(C_{2}T\right)}\varepsilon^{\left(n_{1}T+n_{2}\right)}e^{\left(\frac{I_{1}T+I_{2}}{\varepsilon }\right)}{\dot{\varepsilon }}^{\left(m_{1}T+m_{2}\right)},$$

where: *σ_F_* represents flow stress, MPa; *ε* represents effective strain; $\dot{\varepsilon }$ represents strain rate, s^−1^; *T* represents temperature, °C; and *C_1_*, *C_2_*, *n_1_*, *n_2_*, *I_1_*, *I_2_*, *m_1_*, *m_2_*, are constants presented in Table 2.

In Table 3 presents its chemical composition.

**Figure 2:**
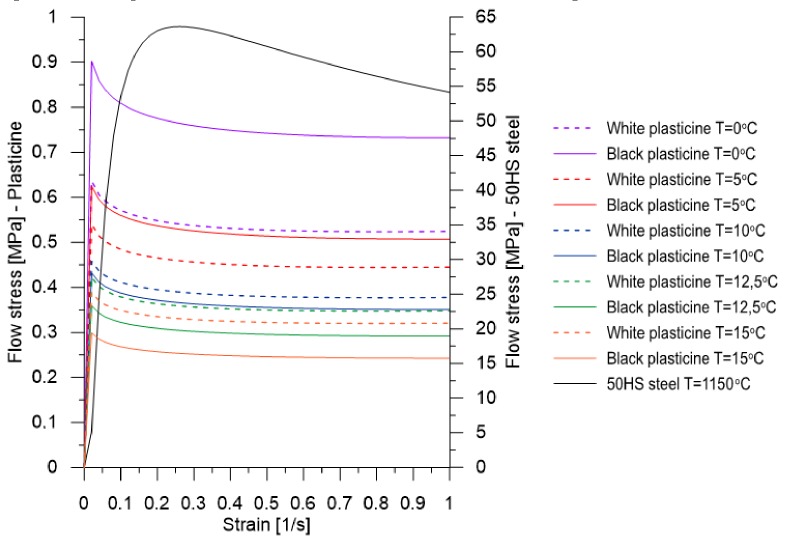
Comparison of material flow curves of commercial plasticine, with 50HS grade steel.

Scheme (Figure 2) presents the comparison of the curves of material flow of plasticine formed in the temperature range 0 °C to 15 °C and 50HS steel formed at the temperature 1150 °C.

Moreover, research on commercial plasticine in regard to the limit value of the Cockcroft-Latham integral (in the tension test) was conducted. The results obtained were then compared to the values obtained for C45 grade steel. A similarity of the limit values of the Cockcroft-Latham criterion was observed for the compared materials. These results were presented in a scientific paper by Wójcik et al. [25]. The limit values of the fracture criterion with black plasticine are presented in Table 4.

Furthermore, tests on determining the damage criterion in the process of rotational compression in a channel of a disc-shaped sample [1,26] were conducted. An analysis of the results has shown a similarity of the behaviour of commercial plasticine and 50HS grade steel formed in the temperature range 950 °C–1150 °C. 

On the basis of the tests on the friction conditions, friction factors and coefficients for the friction couple plasticine—3D ABS print were determined, and the most favourable lubricating agent, allowing one to obtain friction conditions similar to the friction couple hot-formed steel–steel tools [27], was obtained. In the case of cross-wedge rolling processes, the friction occurring is characterized by very significant values of the factors, oscillating in the range 0.8 and 0.9. Upon investigation, it was stated that the friction conditions for plasticine and hot-formed steel are very similar (provided Teflon oil is used as the lubricating agent in the model testing).

## 3. Test Stand and Tools Used in Research

**Figure 3:**
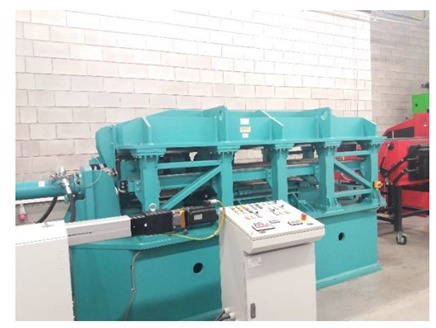
Laboratory cross-wedge rolling mill.

In the laboratory testing of cross-wedge rolling of steel samples, the rolling mill located in the Department of Computer Modelling and Metal Forming Technologies of Lublin University of Technology was used. The rolling mill presented in Figure 3 allows one to install flat wedge tools. During the rolling process, only the upper tool moves, whereas the nether tools remains fixed. The tolling speed is constant at 300 mm/s, whereas the maximum force is 105 kN.

**Figure 4:**
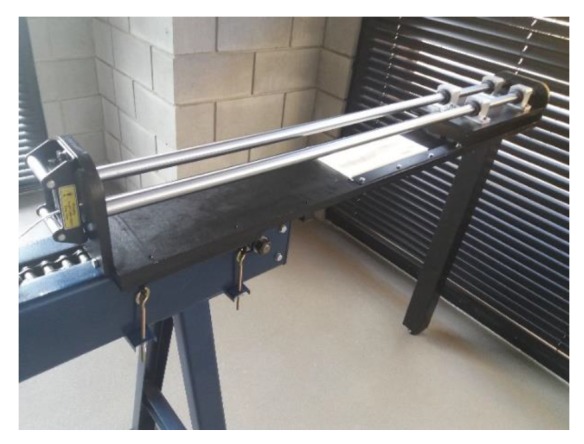
Model cross-wedge rolling mill.

Physical testing was conducted in a model rolling mill, designed and constructed for the research. The lab stand for physical modelling was set up as an additional module of a laboratory chain drawing machine, as shown in Figure 4. The model rolling mill allows one to conduct 1:2 and 1:2.5 scale real tests.

The drawing machine used allows for a smooth regulation of the linear velocity of the tools in the range 0 to 150 mm/s. Additionally the model stand was equipped with a computer station and force measurement sensor AXIS FC1K (AXIS, Gdańsk, Poland) with the maximum measurement range 1 kN. The sensor along with the software enables the measurement of the dynamic force change and registers the results in the form of a spreadsheet. 

**Figure 5:**
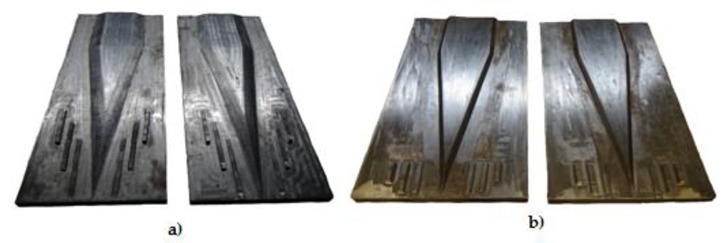
The tools used for testing the limits of cross-wedge rolling of 50HS grade steel in hot working conditions: (**a**) inner cracks (Test 1); (**b**) slippages, neckings and ruptures (Test 2).

The tools used for testing hot-forming of steel (real tests) are shown in Figure 5.

**Figure 6:**
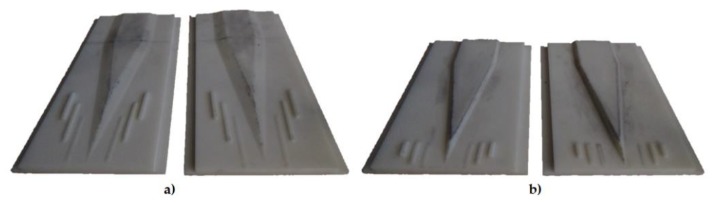
The tools for physical modelling of the limits to the cross-wedge rolling of the model material: (**a**) inner cracks (Test 1); (**b**) slippages, neckings and ruptures (Test 2).

The tools used for physical testing were made of ABS with the 3D printing method. The tools were manufactured using uPrint SE 3D printer produced by Stratasys, based on the FDM (fused deposition modelling). In this method, thin layers of the melted material are applied on top of each other until the model is finished. The tools use for model tests are 1:2.5 scale models on the steel tools, are shown in Figure 6.

The tools for testing the hindering of the inner cracks of the products (Test 1) are 310 × 700 mm. The forming angle α equals 15°, and the β wedge flare angle β = 10°. Such choice of angles ought to initiate the occurrence of cracks along the axis of the formed element. The tools for investigating slippages, neckings and ruptures of the samples (Test 2) are characterised by the forming angle α = 45° and the wedge flare angle 11° 

**Figure 7:**
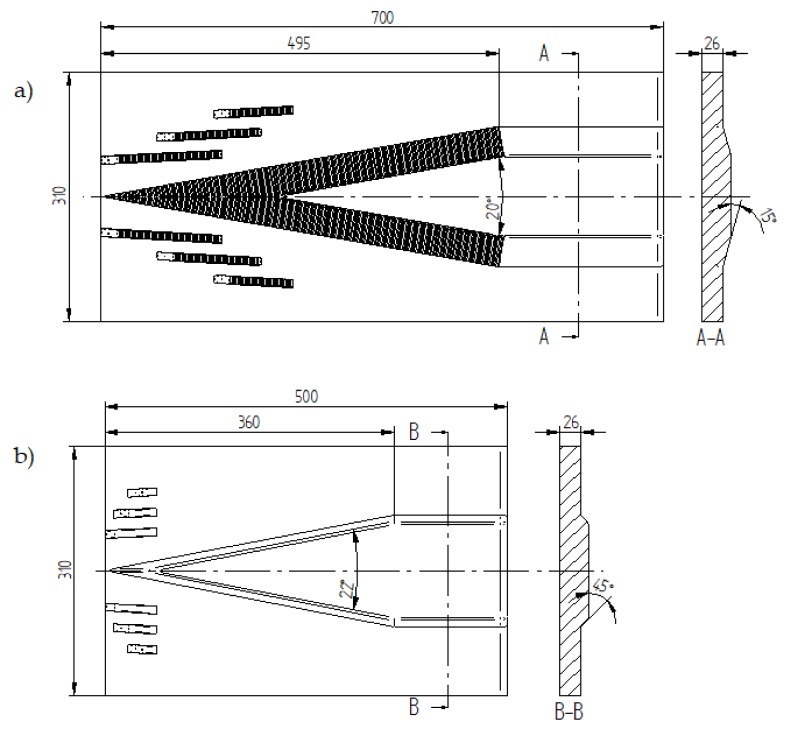
Shape of the tools used for testing and chosen dimensions given: (**a**) tools for testing the inner cracks (Test 1); (**b**) slippages, necking and ruptures (Test 2).

The main dimensions of the tools used are shown in Figure 7.

## 4. Method

For real testing, three samples with various diameters for each stage of the laboratory tests were used. The dimensions of the samples were, respectively, Ø26 × 210 mm, Ø33 × 150 mm and Ø40 × 210 mm (for 50HS grade steel).

The samples used for physical modelling of the cross-wedge rolling were 1:2.5 scale models of the steel balls. The model samples were made of commercial black and white plasticine (three for each diameter). The dimensions of the plasticine samples were Ø10.4 × 84 mm, Ø13.2 × 60 mm and Ø16 × 40 mm.

Upon analysing the papers on physical modelling, an innovative process for preparing the model samples was designed. The method comprises of three main stages.

The first step was hand working of plasticine, pre-heated to 30–35 °C, multiple times in order to eliminate air bubbles created in the production process. The air bubbles have a negative impact on the samples. 

**Figure 8:**
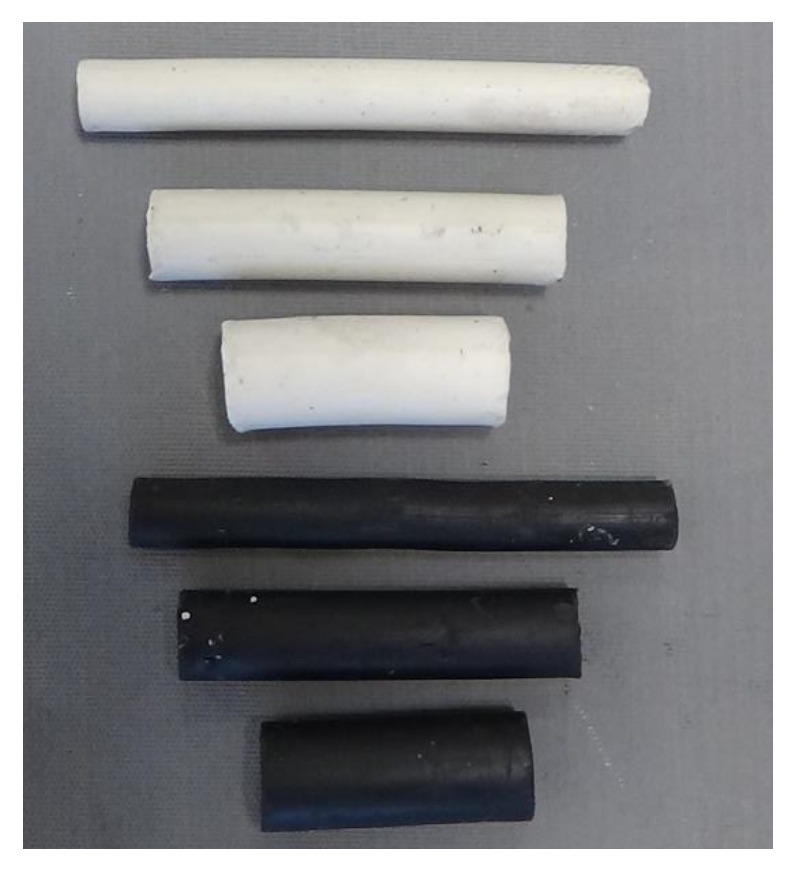
Samples made of the model material.

Another step is extruding rods with a circular cross-section (Ø10.4 mm; Ø13.2 mm; Ø16 mm) from the previously prepared billet. Then, the rod is divided into sections of certain length (84 mm; 60 mm and 40 mm). Figure 8 shows the model samples. 

The last stage of the process was cooling the samples to the forming temperature (0 °C, 5 °C, 10 °C, 12.5 °C and 15 °C) for 24 h. Such a cooling time allows one to obtain a similar temperature in the entire billet.

Physical testing was conducted in a similar manner to the real testing. The only differences were the scales of the models and the formed material. Linear velocity of the model tools was assumed to be 120 mm/s. In order to obtain similar friction conditions between the plasticine and the tools made of ABS, Teflon oil was used. 

## 5. Results

**Figure 9:**
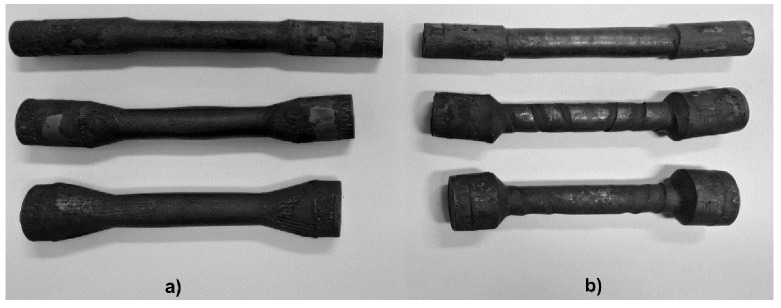
The elements obtained from laboratory testing of the real material using hot-formed 50HS steel: (**a**) test 1, (**b**) test 2.

On the basis of the real and model tests of the cross-wedge rolling, process data allowing one to perform a comparative analysis was gathered. In the first test, the occurrence of cracks inside the finished product was investigated/examined, using three deformation ratios δ = 1.18; δ = 1.5; and δ = 1.81. The inner cracks might result from the incorrect choice of tool parameters, as well as low-cycle material fatigue. The products obtained from test 1 are presented in Figure 9a.

**Figure 10:**
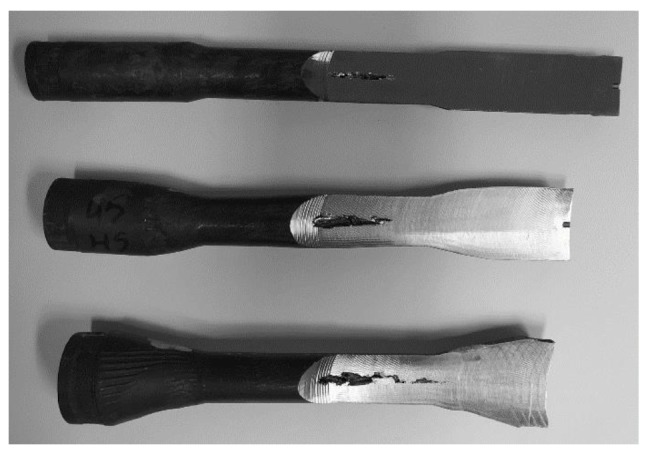
Samples of the first type after milling.

Laboratory tests were conducted thrice and as a result, three samples with varying dimensions were obtained for each test. Then, the samples were milled in order to detect cracks. Figure 10 presents the samples after machining. In each sample, axial cracks of varying diameter and length were observed.

In the samples with the smallest deformation ratio, a crack with a maximum diameter Ø3 mm and length 60 mm occurred. In the samples with the deformation ratio equal δ = 1.5, the diameter and length of the crack were, respectively, Ø4 mm and 70 mm. In the samples with the highest deformation ratio, a crack with a maximum diameter Ø5 mm and length 100 mm occurred.

In Test 2, the aim of the laboratory tests was to examine the limiting phenomena such as slippages, neckings, and ruptures. The real tests were conducted for similar samples and deformation ratios as in Test 1. For the tests, the tools presented in Figure 5b were used. 

The elements obtained in the test are shown in Figure 9b. The samples with the smallest deformation ratio were characterized by the smooth surface of the formed step. During the rolling process, no slippages between the tools and the material were observed. The forgings obtained from the billet with the dimensions Ø33 × 150 mm had neckings in the form of a screw line. During the rolling of a billet with a diameter of Ø33 mm, minor slippages occurred. Forgings rolled from a Ø40 mm had spiral neckings on the forming surface and slippages occurred. The most significant depth of the necking was the same in the cases of rolling with the deformation ratios δ = 1.5 and δ = 1.81 and equalled 0.9 mm.

**Figure 11:**
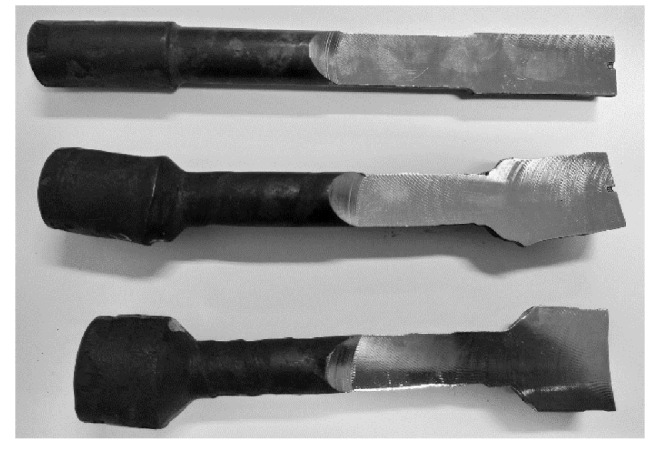
Samples of the second type after milling.

Forgings obtained in this part of the research were also subjected to machining in order to detect cracks and shrinkage porosity inside the sample. The partial sections of the samples are shown in Figure 11.

No limits inside the samples were observed in the machined elements. 

After conducting the tests, the measurements of the diameters and depth of the neckings of the formed steps were taken. The results of the measurements are shown in Table 5.

Another part of the research on the limits to the cross-wedge rolling process was physical modelling using commercial plasticine as the material model for hot-formed 50HS grade steel. The tests were conducted for the material model cooled to five different temperatures: 0 °C, 5 °C, 10 °C, 12.5 °C, and 15 °C.

In the first part of the physical testing, the possibility of modelling the limits that occurring during Test 1 of the real process was investigated, whereas in the second part, the same possibility was investigated for the limits occurring in Test 2. 

**Figure 12:**
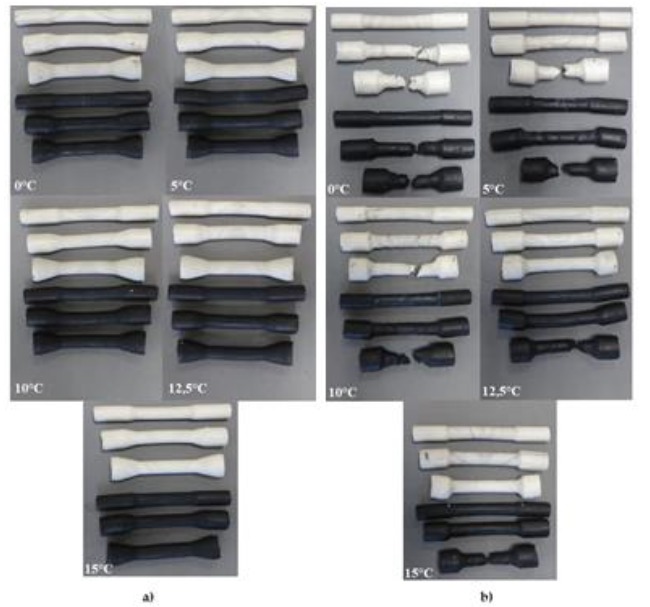
The forgings obtained in the processes of modelling: (**a**) slippages, neckings and ruptures (test 1); (**b**) inner defects (test 2).

The shape of the obtained model forgings is shown in Figure 12.

**Figure 13:**
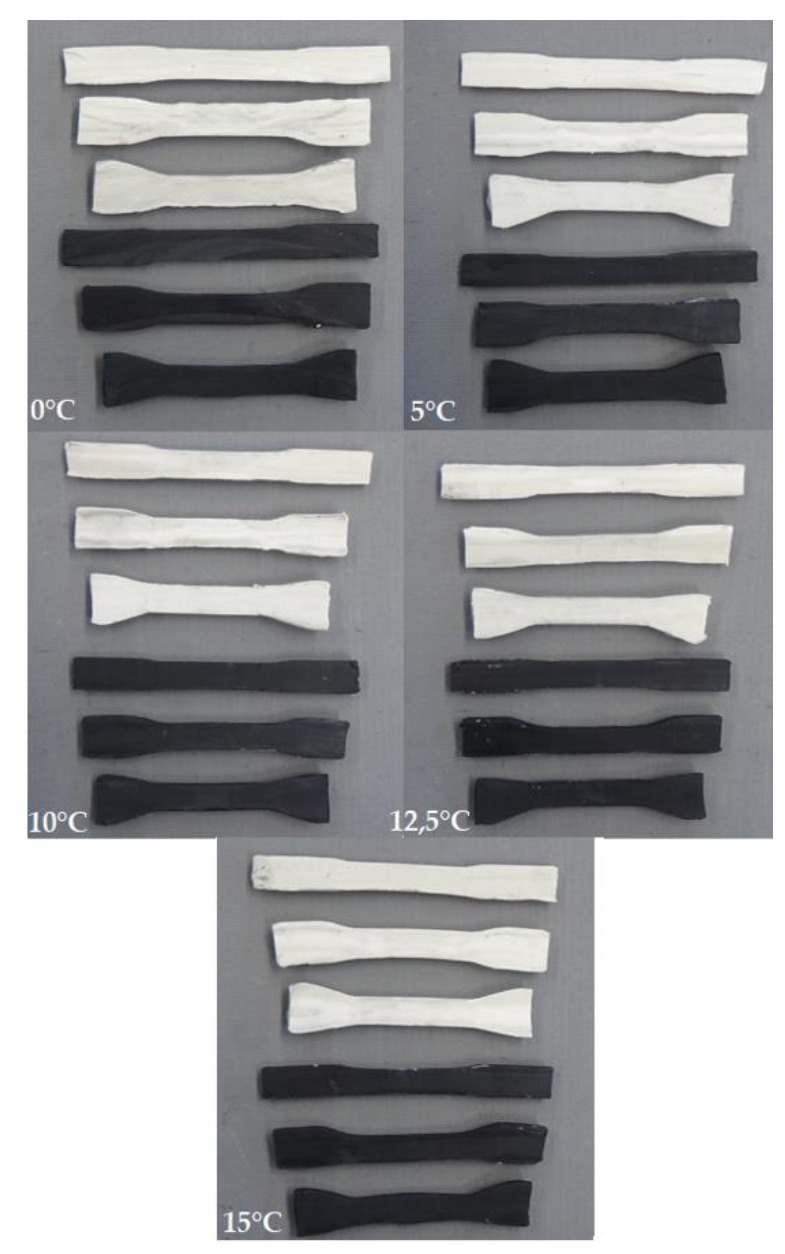
The cross-sections of the samples obtained in the process of modelling the inner defects (type 1).

After conducting the model testing of the first test, the obtained forgings were cut along the axis in order to verify the occurrence of the crackings, as shown in Figure 13. The analysis of the cross-sections of the plasticine elements showed no cracks and shrinkage porosity of the formed material regardless of the forming temperature.

In the second part of the research, a variety of results occurred. The obtained forgings are shown in Figure 12b.

Upon analysing the test results, it was observed that the samples with the smallest diameter were rolled without slippages between the tools and the billet, regardless of the temperature of the material. The samples with the diameter Ø13.2 mm had no ruptures in the majority of cases (the exceptions are white and black samples rolled at 0 °C). The elements made of white plasticine with the biggest diameters were ruptured in the case of cross-wedge rolling of the material cooled to 0 °C, 5 °C and 10 °C, whereas the black samples were ruptured in the entire range of temperatures. The cracks are a result of the significant slippage, which resulted in an immediate rupture of the sample in the beginning of the rolling process. In the samples with medium and the biggest diameter, screw-shaped neckings were observed. The neckings initiated the rupture.

After conducting the tests, the elements were measured using a toolmaker’s microscope. The obtained results are shown in Table 6. 

The average diameter of the step of the formed elements obtained from a Ø10.4 mm billet is equal to 8.87 mm (22.18 mm after scaling), and from a Ø13.2 mm billet is equal to 8.94 mm (after scaling 22.35 mm), whereas for the billet with the biggest diameter, it is equal to 9.18 mm (22.94 mm after scaling). 

As a result of the analysis of the dimensions of the samples obtained in the second test, it was observed that the deepest point of the necking is 0.6 mm, which equals 1.5 mm after scaling. In the samples with the smallest diameter, the maximum depth of the necking was equal to 0.1 mm. The average diameter of the formed step of the element is equal to 8.87 mm (after scaling 22.18 mm) for the diameter Ø13.2 mm and 8.6 mm for the biggest diameter (21.5 mm after scaling).

**Figure 14:**
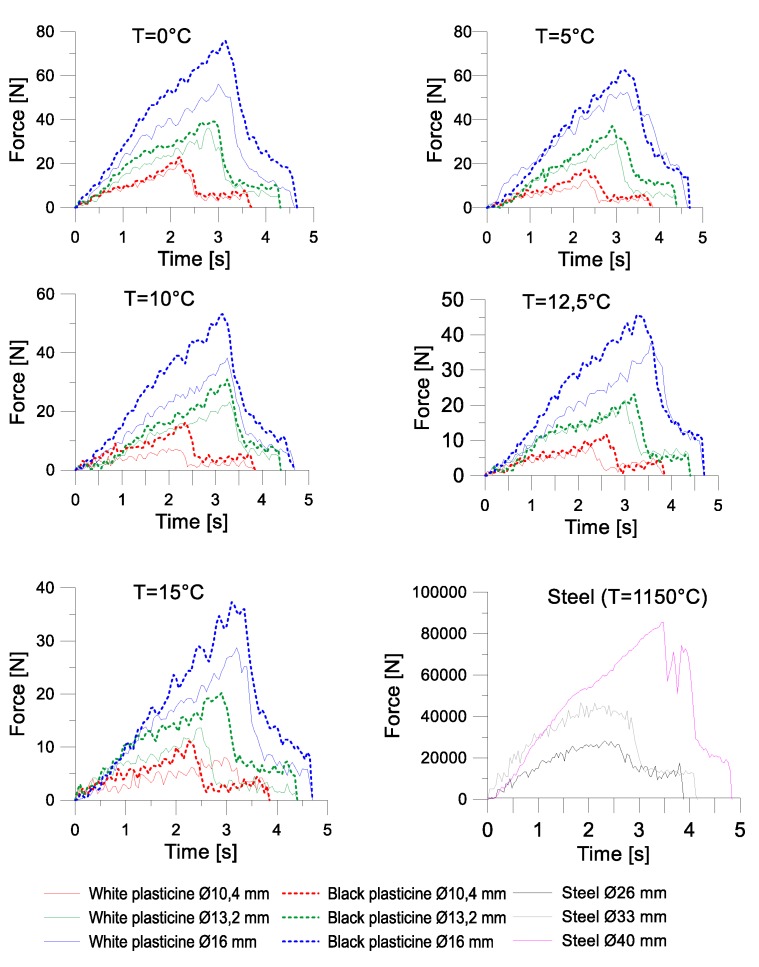
The progress of the forming force in test I.

**Figure 15:**
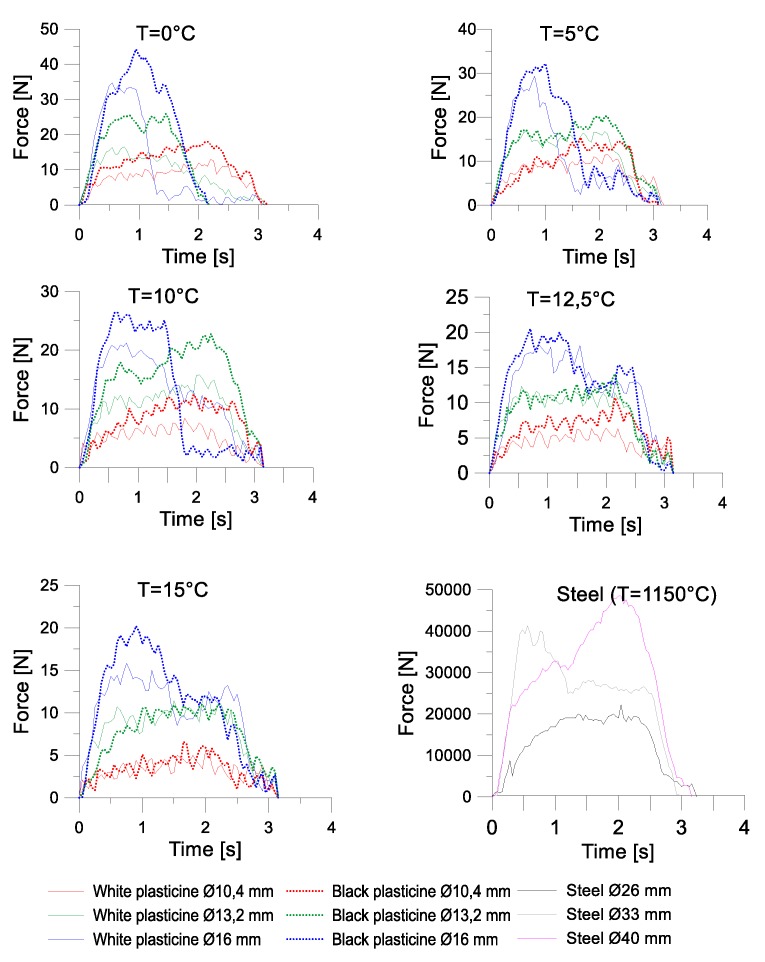
The progress of the forming force in test II.

Figure 14 and Figure 15 present the charts for the forming force occurring during cross-wedge rolling of the model materials and 50HS grade steel. In the case of the first test, the progress of the forming forces in the process of physical modelling is similar to the progress of these forces in the process of rolling steel elements. The values of the forming force increase until the end of the wedge part of the tool. Then, in the calibrating part of the tool, the force drops by over 60%.

Then, calculations aiming to determine the similarity coefficient between the model and real materials at 1150 °C were conducted. The similarity coefficient was calculated on the basis of the dependency (7):

(7) $$\lambda =\;\frac{\int_{0}^{1}\sigma_{F\;steel}d\varepsilon }{\int_{0}^{1}\sigma_{F\;plast}d\varepsilon }\;$$

where: σ_Fsteel_ represents the plastic strain of steel, and σ_Fplast_ represent the plastic strain of plasticine

The values of the obtained similarity coefficients are shown in Table 7. 

After calculating the similarity coefficients, the value of the real forming force was estimated on the basis of Formula (8).

(8) $$F=\lambda \;F^{\prime }s^{2}$$

where: *F*’ represents the forming force from the model testing; λ represents the similarity coefficient of the plasticity of the model material, and s represents the scale of the tools.

The results of the estimations made for the maximum force are shown in Table 8. The comparative analysis of the maximum forces obtained during the second test was conducted using only those sets of samples (three different diameters) in which rupture did not occur.

In Test 1, the greatest similarity of the values of real and model forces was obtained in the process of rolling white and black plasticine at 0 °C, whereas in Test 2, the highest convergency of the maximum value of the force was obtained for white plasticine at 12.5 °C. In the first test, the estimated forming force during model rolling was equal to 12.8 kN for the smallest deformation ratio, 24.5 kN for the deformation ratio δ = 1.5, and 36.3 for the biggest deformation ratio. In test 2, however, the estimated values of force are as follows:

- Deformation ratio δ = 1.18 white plasticine—24.5 kN, black plasticine—28.7 kN;
- Deformation ratio δ = 1.5 white plasticine—41.6 kN, black plasticine—51.9 kN;
- Deformation ratio δ = 1.81 white plasticine—80.1 kN, black plasticine—81 kN.

Black plasticine required higher forces in the forming process than white plasticine. The most significant difference was observed for deformation ratio δ = 1.5, equalling 24%. The smallest difference between the forces obtained in real and physical testing was 1% in the case of the biggest deformation ratio. 

## 6. Conclusions

The comparison of cross-wedge rolling of elements made of 50HS grade steel and physical modelling allowed us to determine the applicability of plasticine as a material model for simulating the limits to the rolling process. 

Samples made of black plasticine cannot be used to model such limiting phenomena as inner cracks due to the lack thereof in the conducted tests as well as the instant rupture at every temperature. As far as white plasticine is concerned, it can model the limiting phenomena occurring during the hot-forming process of 50HS grade steel only in the case of temperatures of 12.5 and 15 °C. The values of the estimated force obtained for 12.5 °C were more similar to the ones from the real process.

The diameters of the rolled billet in test 1 were best modelled by white plasticine at 12.5 °C: The diameter of the plasticine samples was equal to Ø 22.2 ± 0.5 mm, whereas for the steel samples, it was equal to Ø 22.5 ± 0.1 mm.

The diameters of the rolled element in Test 2 were best modelled with white and black plasticine at 0 °C: The diameter of the plasticine samples equals Ø 21.8 ± 0.2 mm, whereas in the case of the steel samples, it equals Ø 21,85 ± 0,05 mm. Modelling the depth of the necking was as follows: physical modelling—0.75 mm; FEM simulation—0.85 mm; real rolling—0.9 mm. 

The tests showed that physical modelling of the first test allowed one to estimate the forces smaller, on average, by 9.3% than in the real process; whereas in the model testing of test 2, the observed forces were smaller by 35.7%. 

## Figures and Tables

**Table 1:** Constant of the white and black plasticine material models.

Material	C1	n1	n2	m	b	a
White plasticine	0.48057	−0.0313	0.08705	0.2451	−0.0026	−0.03283
Black plasticine	0.6817	−0.0711	0.07203	0.2701	−0.0037	−0.07358

**Table 2:** Constant of the 50HS steel material model. (DEFORM—user guide).

Material	C_1_	C_2_	n_1_	n_2_	I_1_	I_2_	m_1_	m_2_
50HS	6086.5	−0.0041	−0.00037	0.155	0.000013	−0.085	−0.000008	0.14

**Table 3:** Percentage chemical compositions used for testing material.

Material	C	Mn	Si	P max	S max	Cr max	Ni max	Cu max
50HS	0.45 – 0.55	0.3 – 0.6	0.8 – 1.2	0.03	0.03	0.9 – 1.2	0.4	0.25

**Table 4:** Limit values of the Cockcroft-Latham integral determined for plasticine PRIMO [25].

Temperature [°C]	Material	Limit Values of the Cockcroft-Latham Integral
0	White plasticine	0.646
	Black plasticine	0.691
5	White plasticine	0.786
	Black plasticine	1.34
10	White plasticine	1.27
	Black plasticine	1.25
15	White plasticine	1.38
	Black plasticine	2.04
20	White plasticine	1.45
	Black plasticine	2.00
900	C45 steel	0.849
1000	C45 steel	0.682
1100	C45 steel	0.765
1200	C45 steel	0.726

**Table 5:** The measurements of diameters and depths of the necking with 50HS grade steel.

Temperature [°C]	Diameter [mm]	Davg [mm]		Davg [mm]	Max Depth of Necking [mm]
		Test 1		Test 2	
1150	Ø26	22.4	21.8	22.1	-
	Ø33	22.5	21.9	21.5	0.9
	Ø40	22.6	21.9	20.6	0.9

**Table 6:** The dimensions of the model samples after cross-wedge rolling.

T [°C]	Material	Sample Diameter [mm]	Test 1	Test 2	
			D_I avg_ (Scale 2.5) [mm]	D_II avg_ (Scale 2.5) [mm]	Depth Necking (Scale 2.5) [mm]
0	White plasticine	Ø10.4	8.76	8.75	0.1
		Ø13.2	9.00	8.88	0.3
		Ø16	9.19	8.4	Crack
	Black plasticine	Ø10.4	8.87	8.9	0.1
		Ø13.2	9.09	8.68	0.3
		Ø16	9.29	8.9	Crack
5	White plasticine	Ø10.4	8.745	8.89	0.1
		Ø13.2	9.025	8.88	0.3
		Ø16	9.21	8.6	Crack
	Black plasticine	Ø10.4	8.86	8.99	0.4
		Ø13.2	8.94	9.03	0.4
		Ø16	9.26	8.5	Crack
10	White plasticine	Ø10.4	8.67	8.81	0.1
		Ø13.2	8.94	8.73	0.6
		Ø16	9.06	8.98	0.5 (crack)
	Black plasticine	Ø10.4	8.80	9.00	0.1
		Ø13.2	8.92	8.88	0.3
		Ø16	9.23	8.80	Crack
12.5	White plasticine	Ø10.4	8,72	8,57	0,05
		Ø13.2	8.87	8.72	0.3
		Ø16	9.12	8.5	0.35
	Black plasticine	Ø10.4	8.79	8.76	0.1
		Ø13.2	9.06	8.91	0.05
		Ø16	9.22	8.70	0.15 (crack)
15	White plasticine	Ø10.4	8.69	8.85	0.1
		Ø13.2	8.72	9.04	0.5
		Ø16	9.06	8.01	0.35
	Black plasticine	Ø10.4	8.84	8.73	0.1
		Ø13.2	8.83	8.92	0.05
		Ø16	9.12	8.68	0.5 (crack)

**Table 7:** Similarity coefficients for model material and 50HS grade steel.

Temperature [°C]	Material	Similarity Factor λ Steel 50HS T = 1150 °C
0	White plasticine	228
	Black plasticine	171
5	White plasticine	260
	Black plasticine	237
10	White plasticine	298
	Black plasticine	329
15	White plasticine	298
	Black plasticine	329
20	White plasticine	319
	Black plasticine	387

**Table 8:** Estimated forces of model rolling and the real process [kN].

	Temperature [°C]	White Plasticine			Black Plasticine			Steel 50HS T = 1150 °C		
		Ø10.4	Ø13.2	Ø16	Ø10.4	Ø13.2	Ø16	Ø26	Ø33	Ø40
Test 1	0	24.5	41.6	80.1	28.7	51.9	81.0	28.2	46.7	85.5
	5	20.6	49.7	85.3	26.1	55.1	92.6			
	10	18.6	43.2	71.1	33.3	63.5	109.4			
	12.5	16.3	42.9	75.0	28.3	55.9	110.3			
	15	16.2	27.5	58.2	38.7	70.5	130.0			
Test 2	0	18.4	23.5	49.4	19.2	27.7	47.2	22.2	41.2	48.5
	5	18.7	27.3	47.6	22.8	35.8	47.7			
	10	15.6	29.4	39.5	25.5	46.9	54.3			
	12.5	12.8	24.5	36.3	25.9	34.1	49.6			
	15	11.9	25.6	33.7	18.8	31.3	57.4			

## References

[1] Wójcik Ł., Pater Z. (2019). Phisical simulation of the Mannesmann effect in the rolling process. Arch. Metall. Mater..

[2] Pater Z. (2009). Walcowanie Poprzeczno-Klinowe.

[3] Bartnicki J., Pater Z. (2005). Walcowanie Poprzeczno—Klinowe Wyrobów Drążonych.

[4] Andreev G.V., Guzjavičus L.V., Makušok E.M., Ščukin V.J. (1975). Vybor geometričeskich parametrov klinovogo instrumenta. Abrazivnaja Obrabotka i Obrabotka Metallov Rezaniem i Davlenem.

[5] Pater Z., Samołyk G. (2013). Podstawy Technologii Obróbki Plastycznej Metali.

[6] Liu G., Zhong Z., Shen Z. (2014). Influence of reduction distribution on internal defects during cross wedge-rolling process. Procedia Eng..

[7] Dong Y., Tagavi K.A., Lovell R.A., Deng Z. (2000). Analysis of stress in cross wedge rolling with application to failure. Int. J. Mech. Sci..

[8] Liu G., Ren G., Xu C., Jiang Z., Shen Z. (2004). Research on mechanism of interior-hollow defect during the defermation of cross wedge rolling. J. Mech. Eng..

[9] Fang G., Lei L.P., Zeng P. (2002). Three-dimensional rigid–plastic finite element simulation for the two-roll cross-wedge rolling process. J. Mater. Process. Technol..

[10] Ghiotti A., Fanini S., Bruschi S., Bariani P.F. (2009). Modelling of the Mannesmann effect. CIRP Ann.—Manuf. Technol..

[11] Pater Z., Tomczak J., Bulzak T., Bartnicki J., Tofil A. (2019). Prediction of Crack Formation for Cross Wedge Rolling of Harrow Tooth Preform. Materials.

[12] Bulzak T., Pater Z., Tomczak J. (2017). Numerical and experimental analysis of a cross wedge rolling process for producing ball studs. Arch. Civ. Mech. Eng..

[13] Pater Z., Tofil A., Tomczak J., Bulzak T. (2015). Numerical analysis of the cross wedge rolling process (CWR) for a stepped shaft. Metalurgija.

[14] Kowalczyk L. (1995). Modelowanie Fizykalne Procesów Obróbki Plastycznej.

[15] Chijiwa K., Hatamura Y., Hasegawa N., Tanabe Y. (1984). Simulation of horizontal 2-stands rolling by plasticine. Trans. Iron Steel Inst. Jpn..

[16] Mandic V., Stefanovic M. (2002). Physical modelling and FEM simulation of the hot bulk forming processes. J. Technol. Plast..

[17] Zhan M., Liu Y., Yang H. (2001). Physical modeling platform using plasticine. J. Mater. Process Technol..

[18] Balasundar I., Sudhakara M., Raghu T. (2009). Equal channel angular pressing die to extrude a variety of materials. Mater. Des..

[19] Khalili Meybodi A., Assempour A., Farahani S. (2012). A general methodology for bearing design in non symmetric T—Shaped sections in extrusion process. J. Mater. Process. Technol..

[20] Danno A., Tanaka T. (1984). Hot forming of stepped steel shafts by wedge rolling with three rolls. J. Mech. Work. Technol..

[21] Dong Y., Lovell M., Tagavi K. (1998). Analysis of interfacial slip in cross-wedge rolling: An experimentally verified finite-element model. J. Mater. Process. Technol..

[22] Wójcik Ł., Pater Z. (2017). Physical Analysis of Cross-Wedge Rolling Process of a Stepped Shaft. Adv. Sci. Technol. Res. J..

[23] Pater Z., Tomczak J., Wójcik Ł., Bulzak T. (2019). Physical modelling of the ball-rolling processes. Metals.

[24] Wójcik Ł., Lis K., Pater Z. (2016). Plastometric tests for plasticine as physical modelling material. Open Eng..

[25] Wójcik Ł., Pater Z. (2018). Comparison analysis of cockroft - Latham criterion values of commercial plasticine and C45 steel. Acta Mech. Autom..

[26] Pater Z., Walczuk P., Lis K., Wójcik Ł. (2018). Preliminary analysis of a rotary compression test. Adv. Sci. Technol. Res. J..

[27] Wójcik Ł., Pater Z. (2018). Wpływ temperatury i smarowania na współczynnik pary trącej plastelina handlowa - kopolimer abs. Hut.—WIADOMOŚCI Hut..

